# Supplementary figures and images for: A noninvasive BCG skin challenge model for assessing tuberculosis vaccine efficacy
Source: PLoS Biol. 2024 Aug 19;22(8):e3002766. doi: 10.1371/journal.pbio.3002766 (PMC11361749; doi:10.1371/journal.pbio.3002766)

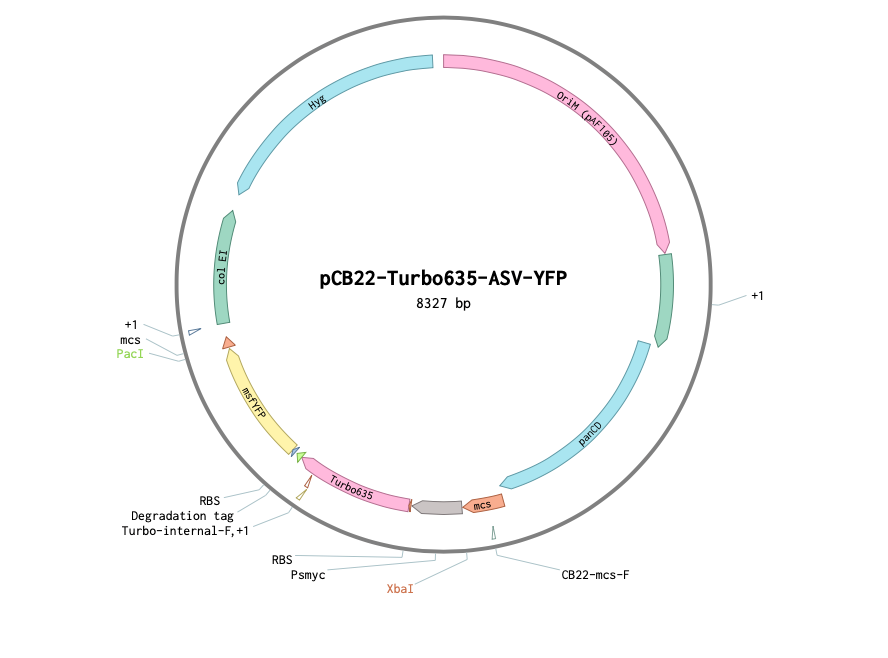

Supplement: S1 Fig — (TIF) [file pbio.3002766.s001.tif]

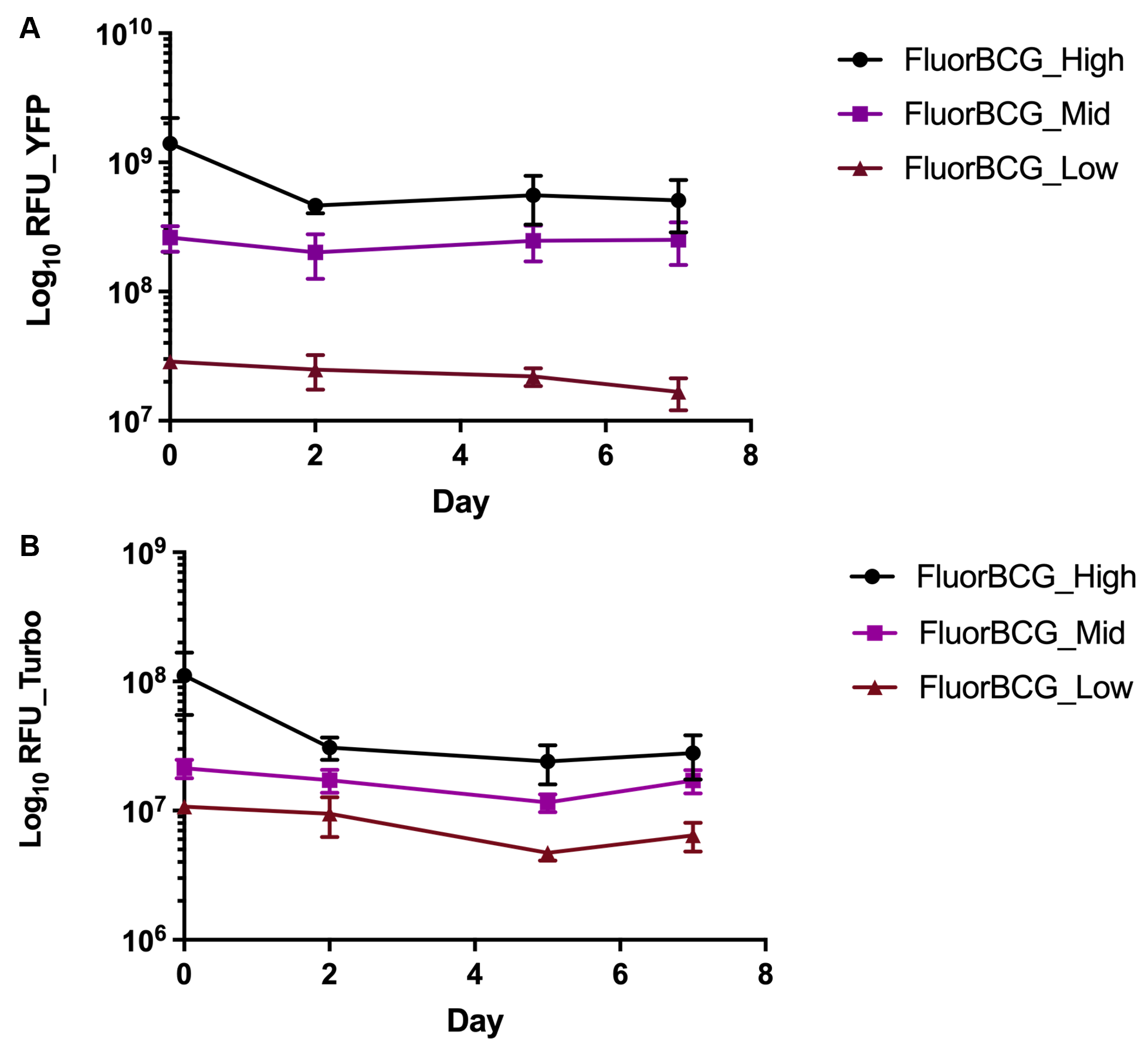

Supplement: S2 Fig — BALB/c mice were infected ID with 3 concentrations of FluorBCG: FluorBCG_High of 5 × 10^6^ CFU, FluorBCG_Mid of 5 × 10^5^ CFU, and FluorBCG_Low of 5 × 10^4^ CFU. (A) Raw YFP fluorescence. (B) Raw Turbo-635 fluorescence from unvaccinated mice. Data represent mean fluorescence ± SD from n = 3 mice (mean of 2 ears per mouse). The data underlying this figure can be found in S1 Data. BCG, bacille Calmette-Guérin; CFU, colony-forming unit; ID, intradermally; YFP, yellow fluorescent protein. (TIF) [file pbio.3002766.s002.tif]

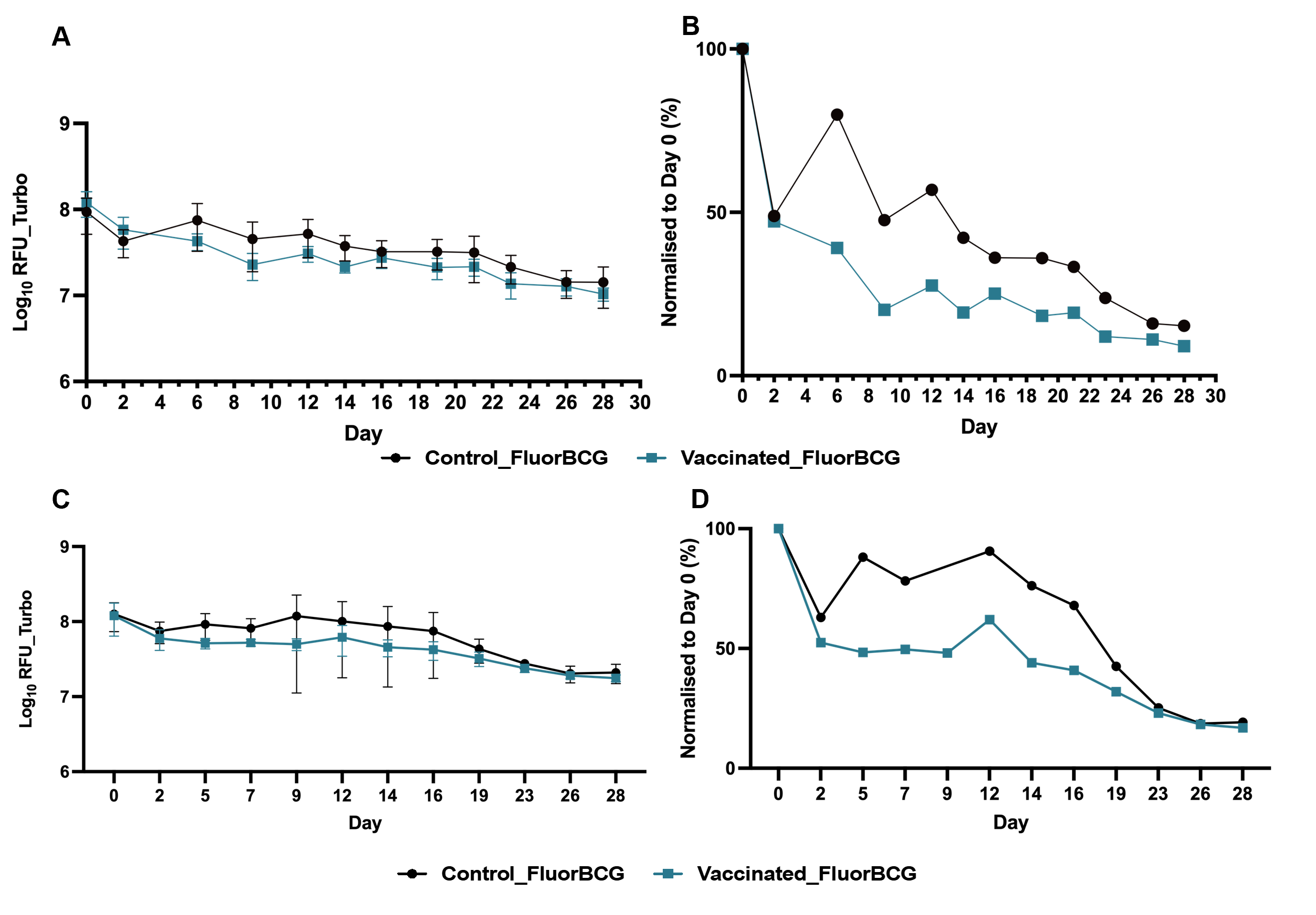

Supplement: S3 Fig — (A, C) Raw Turbo-635 fluorescence. (B, D) Normalised Turbo-635 fluorescence from both control and vaccinated mice post-ID skin challenge with fluorescent BCG. Representative data from one of 2 experiments are shown. Data represent mean fluorescence ± SD from n = 5 mice (average of 2 ears per mouse). The data underlying this figure can be found in S1 Data. (TIF) [file pbio.3002766.s003.tif]

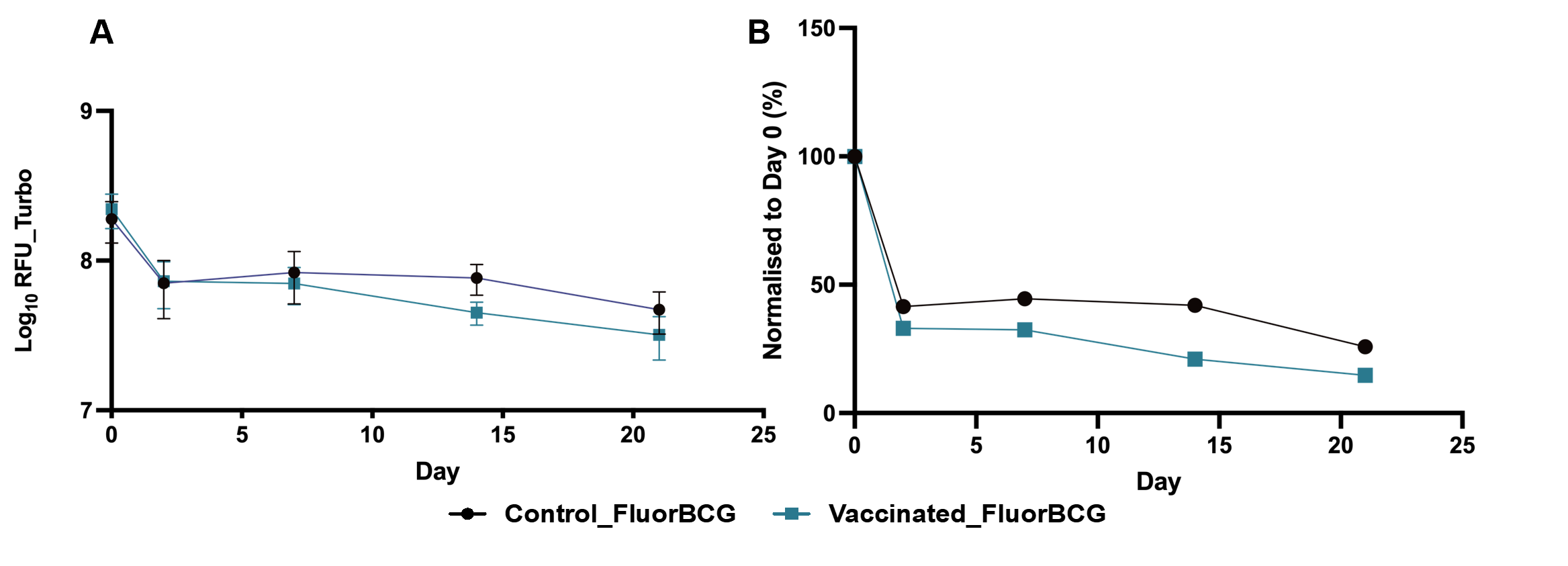

Supplement: S4 Fig — Mice were imaged and fluorescence from (A) Turbo-635 channels were quantified and normalised to day 0 (B) following intradermal skin challenge with fluorescent BCG. Data from one of 2 experiments are shown representing mean fluorescence ± SD from n = 5 mice. The data underlying this figure can be found in S1 Data. (TIF) [file pbio.3002766.s004.tif]

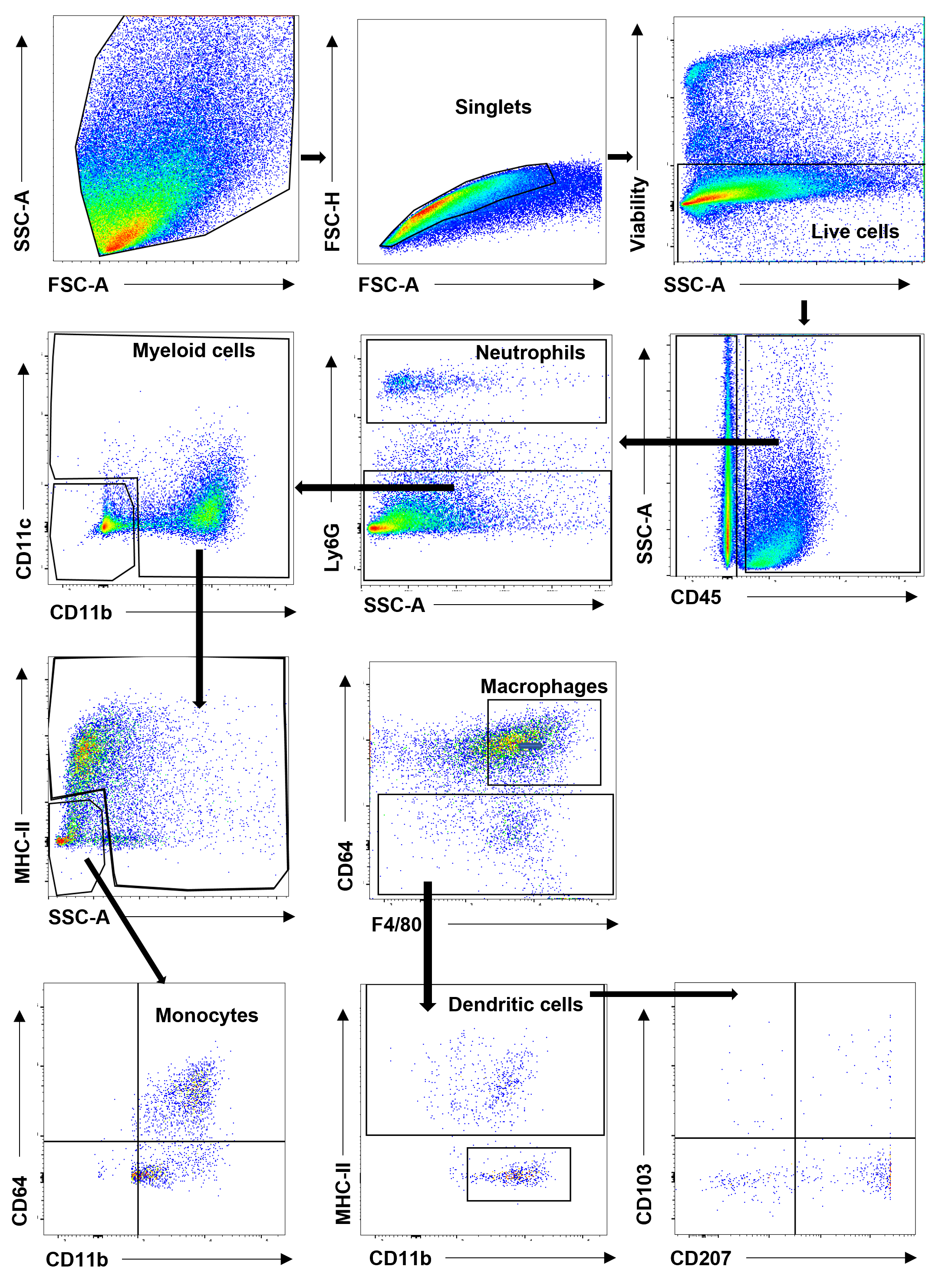

Supplement: S5 Fig — Total cells were isolated from murine ear, and single cells were identified using the FSC-A and FSC-H plot. The viability of the skin cell suspension was determined using the SSC-A and viability plot. Viable cells were gated to identify the CD45+cells and then neutrophils (CD45+Ly6G+), dendritic cells subdivided into Langerhans cells* (CD45+MHC-II+CD207+CD103−), dermal DCs (CD45+MHC-II+CD207−CD103+) and dermal langerin+ DCs (CD45+MHC-II+CD207+CD103+); monocytes (CD45+CD11b+CD64int) and macrophages (CD45+MHC-II+F4/80+CD64+). (TIF) [file pbio.3002766.s005.tif]

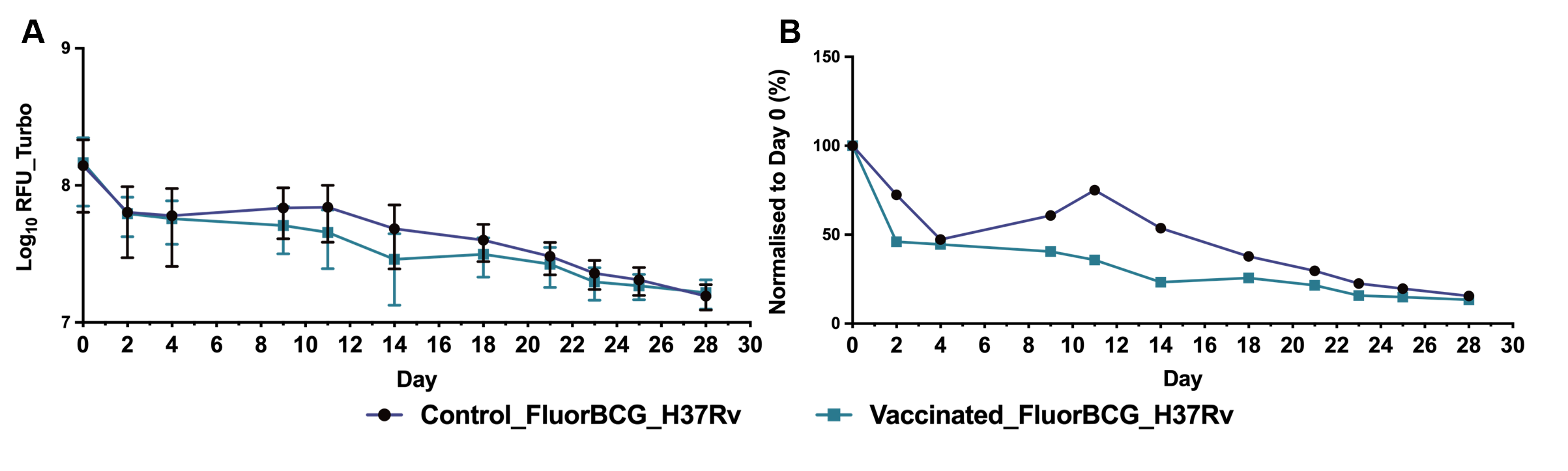

Supplement: S6 Fig — BALB/c mice were immunised with a single dose of BCG, and, after a 4-week rest period, mice were challenged ID in the ear with 5 × 10^6^ CFU of FluorBCG and IN with 1 × 10^3^ H37Rv (FluorBCG+H37Rv). Fluorescence intensities from the site of ID challenge in the ear are represented as raw outputs from the Turbo-635 channel (A) and fluorescence values normalised to day 0 (B). Representative data from duplicate experiments are displayed. Data represent mean ± SD from n = 5 mice. The data underlying this figure can be found in S1 Data. BCG, bacille Calmette-Guérin; CFU, colony-forming unit; ID, intradermally; IN, intranasally. (TIF) [file pbio.3002766.s006.tif]

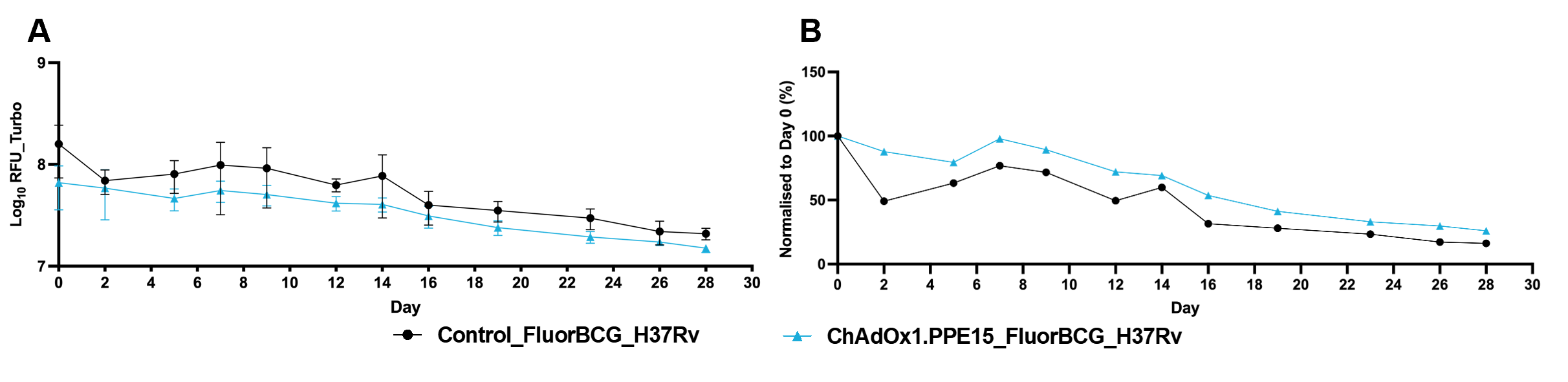

Supplement: S7 Fig — BALB/c mice were immunised with a single dose of ChAdOx1.PPE15, and, after a 4-week rest period, mice were challenged ID in the ear with 5 × 10^6^ CFU of FluorBCG and IN with 1 × 10^3^ H37Rv (FluorBCG+H37Rv). Fluorescence intensities from the site of ID challenge in the ear are represented as raw outputs from the Turbo-635 channel (A) and fluorescence values normalised to day 0 (B). Representative data from duplicate experiments are displayed. Data represent mean ± SD from n = 5 mice. The data underlying this figure can be found in S1 Data. BCG, bacille Calmette-Guérin; CFU, colony-forming unit; ID, intradermally; IN, intranasally. (TIF) [file pbio.3002766.s007.tif]

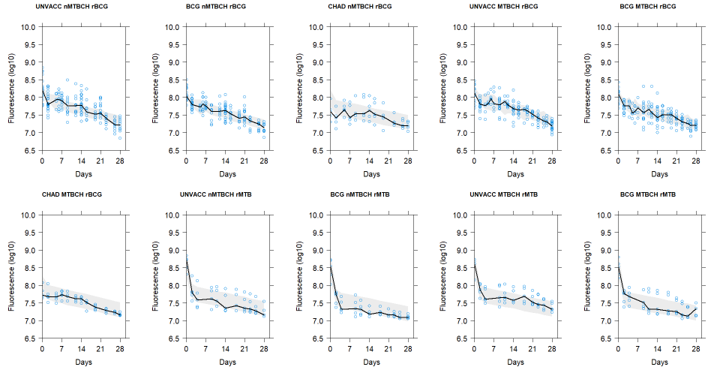

Supplement: S8 Fig — The solid line is the median of the observed data, the shaded area is the 95% confidence interval for the median, and the open blue circles are the observations. The VPC was generated in NONMEM using the PsN VPC command. It can be regenerated by utilising the software specified in the statistical analysis method section and the S2 Data. (TIF) [file pbio.3002766.s008.tif]

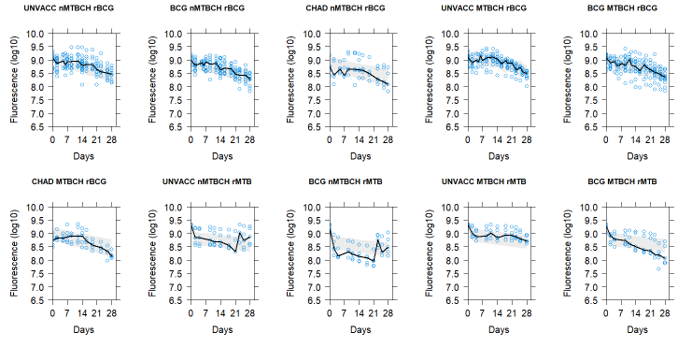

Supplement: S9 Fig — The solid line is the median of the observed data, the shaded area is the 95% confidence interval for the median, and the open blue circles are the observations. The VPC was generated in NONMEM using the PsN VPC command. It can be regenerated by utilising the software specified in the statistical analysis method section and the S3 Data. (TIF) [file pbio.3002766.s009.tif]

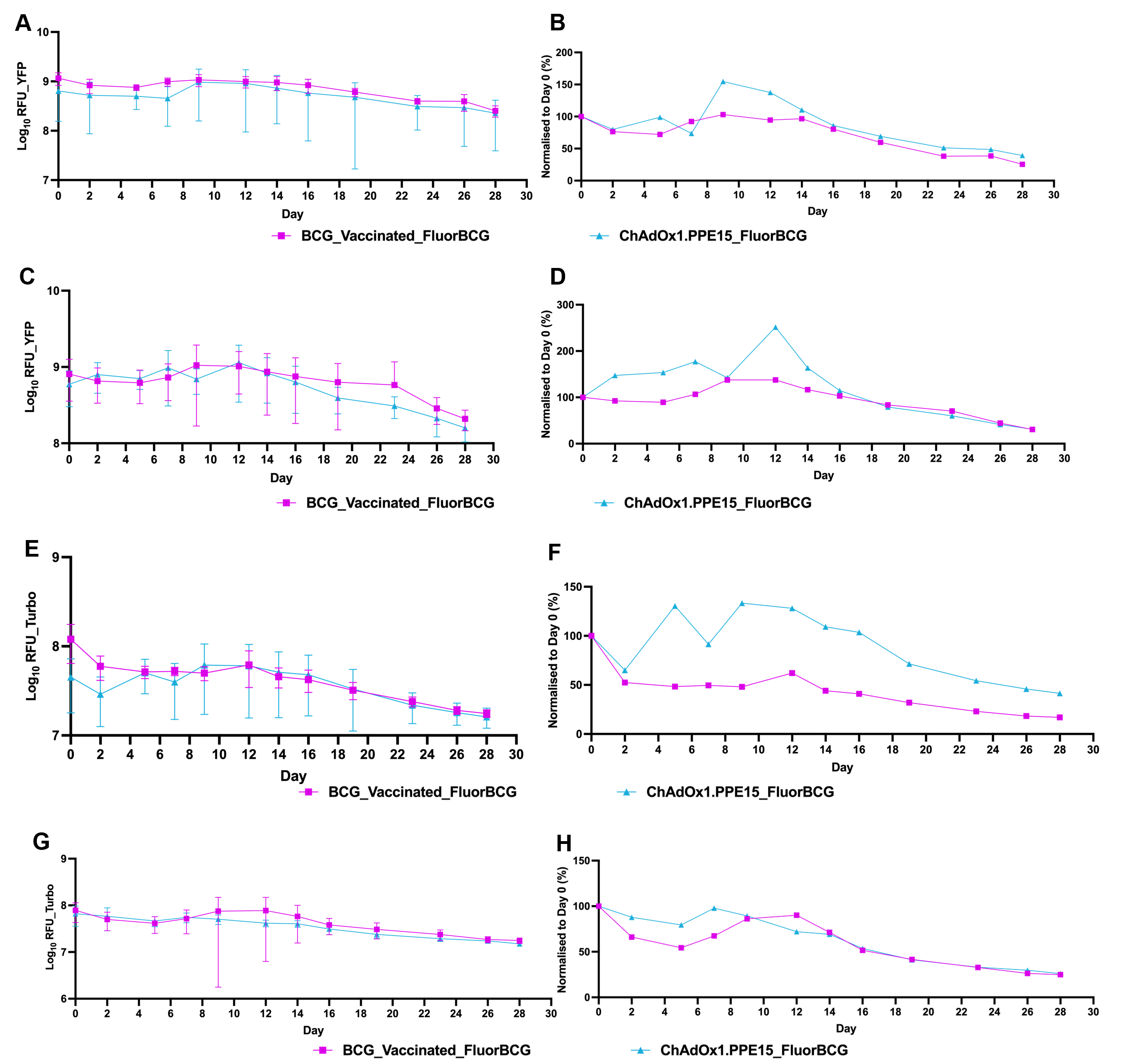

Supplement: S10 Fig — BALB/c mice were either immunised with a single dose of BCG or a single intranasal dose of ChAdOx1.PPE15, and, after a 4-week rest period, mice were challenged ID in the ear with 5 × 10^6^ CFU of FluorBCG. (A, C) Raw YFP fluorescence. (E, G) Raw Turbo-635 fluorescence. (B, D) Normalised YFP fluorescence from both BCG- and ChAdOx.PPE15-vaccinated mice post-ID skin challenge with fluorescent BCG (F, H) Normalised Turbo-635 fluorescence from both BCG- and ChAdOx.PPE15-vaccinated mice post-ID skin challenge with fluorescent BCG. Data represent the mean fluorescence ± SD from n = 5 mice (an average of 2 ears per mouse). The data underlying this figure can be found in S1 Data. BCG, bacille Calmette-Guérin; CFU, colony-forming unit; ID, intradermally; YFP, yellow fluorescent protein. (TIF) [file pbio.3002766.s010.tif]

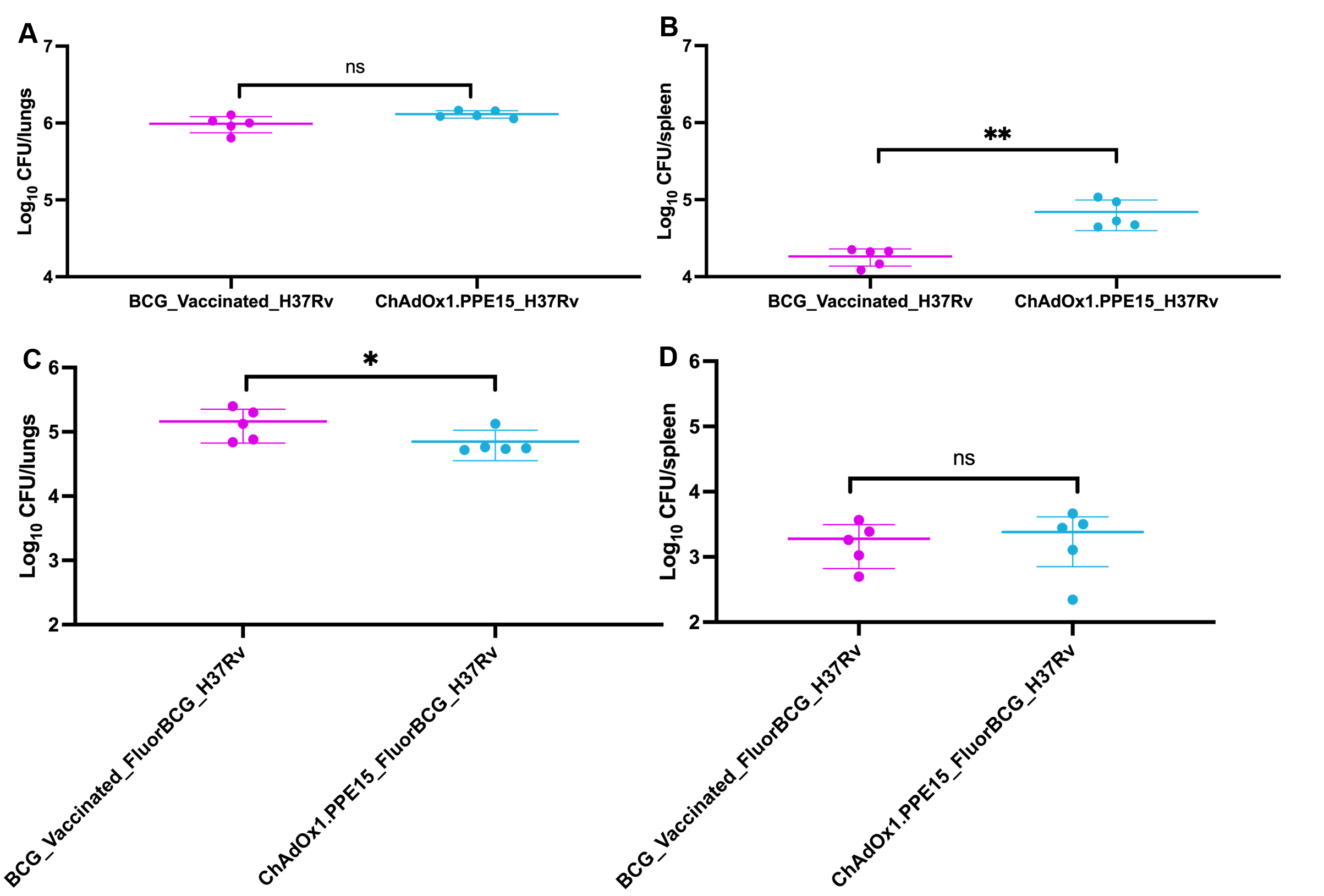

Supplement: S11 Fig — BALB/c mice were either immunised with a single dose of BCG or a single intranasal dose of ChAdOx1.PPE15, and, after a 4-week rest period, mice were either challenged ID in the ear with 5 × 10^6^ CFU of FluorBCG and challenged IN with 1 × 10^3^ H37Rv (H37Rv) or only challenged with 1 × 10^3^ H37Rv (H37Rv). Lungs (A, C) and spleen (B, D) were harvested 4 weeks post-challenge and processed to quantify the bacterial burden. Data represent mean ± SD from n = 5 mice. * p > 0.05, ** p > 0.01. The data underlying this figure can be found in S1 Data. BCG, bacille Calmette-Guérin; CFU, colony-forming unit; ID, intradermally; IN, intranasally. (TIF) [file pbio.3002766.s011.tif]
