# Supplementary material for: A noninvasive BCG skin challenge model for assessing tuberculosis vaccine efficacy
Source: PLoS Biol. 2024 Aug 19;22(8):e3002766. doi: 10.1371/journal.pbio.3002766 (PMC11361749; doi:10.1371/journal.pbio.3002766)
Supplement: S1 Table — Each experiment had 5 mice per group and was measured for both ears; mean ears are tabulated. VC-2, VC-4, and VC-5 had 12 RFU readings taken over 28 days. Flow1 and Flow2 had 5 RFU readings taken over 21 days and were also used for flow cytometry and bacterial load measurements in ears and lymph nodes. (DOCX) [file pbio.3002766.s012.docx]

**Table S1 Experimental Summary**

| **Experimental treatments** | | | **Experiment name** | | | | |
| --- | --- | --- | --- | --- | --- | --- | --- |
| **Vaccine** | **Reporter** | **Mtb challenge** | **VC-2** | **VC-4** | **VC-5** | **Flow1** | **Flow2** |
| Unvacc | Fluor-BCG | No | X |  | X | X | X |
| BCG | Fluo-rBCG | No | X |  | X | X | X |
| Unvacc | Fluor-BCG | H37Rv | X | X | X |  |  |
| BCG | Fluor-BCG | H37Rv | X | X | X |  |  |
| Unvacc | No | H37Rv | X | X | X |  |  |
| BCG | No | H37Rv | X | X | X |  |  |
|  |  |  |  |  |  |  |  |
| Unvacc | Fluor-TB | No |  | X |  |  |  |
| BCG | Fluor-TB | No |  | X |  |  |  |
| Unvacc | Fluor-TB | H37Rv |  | X |  |  |  |
| BCG | Fluor-TB | H37Rv |  | X |  |  |  |
|  |  |  |  |  |  |  |  |
| ChAdOx1.PPE15 | Fluor-BCG | No |  |  | X |  |  |
| ChAdOx1.PPE15 | Fluor-BCG | H37Rv |  |  | X |  |  |
| ChAdOx1.PPE15 | No | H37Rv |  |  | X |  |  |

Each experiment had 5 mice per group and was measured for both ears; mean ears are tabulated.

VC-2, VC-4 and VC-5 had 12 RFU readings taken over 28 days.

Flow1 and Flow2 had 5 RFU readings taken over 21 days and were also used for Flow cytometry and bacterial load measurements in ears and lymph nodes.
