# Supplementary material for: A noninvasive BCG skin challenge model for assessing tuberculosis vaccine efficacy
Source: PLoS Biol. 2024 Aug 19;22(8):e3002766. doi: 10.1371/journal.pbio.3002766 (PMC11361749; doi:10.1371/journal.pbio.3002766)
Supplement: Attachment — Submitted filename: Reviewer Response 26Apr24.docx [file pbio.3002766.s016.docx]

Changes are highlighted in yellow TB_CHIM_manuscript_highlight 26Apr24

**Reviewer #1:**

The authors describe a present the pre-clinical evaluation of a new tool (fluorescent BCG) for human challenge experiments.  The idea and the tool are meritorious, but the presentation of the findings made it a bit challenging to extract the key messages.

1) Abstract:  Starts with human challenge model on line 12.  I was expecting human data.  Line 25: vaccinated mice.  Shouldn't it be stated somewhere in the methods section that this is a mouse study?

**We have mentioned earlier on line 18 that this is characterisation of our model in the mouse.**

2) Overall experimental plan:  There were at least two studies presented, that were partially overlapping.  As I read it, they asked two questions: How good is this non-invasive tool as a function of BCG or subunit vaccination?  Where are the live bacteria in the mouse ears using FACS?  I understand the direct relevance of the first question for human challenges.  I do not see the link between the second question and human challenges.  If there is an anticipated use case in giving BCG to human volunteers and then cutting out the site of BCG challenge for FACS, this should be stated.  This seems to go against the other goal, which is non-invasive monitoring.

**The primary goal of the study was to develop and characterise a non-invasive imaging method for following vaccine effects. We took the opportunity presented by the mouse model to use FACS to also investigate the immune cell subsets involved in the ear, and look at bacterial load, but this would not be a part of an optimised human study.**

3) Statistical analysis section was 2 pages, but in the end, we don't see any data or statistics (p-values or confidence intervals) in the abstract or figures or figure legends.  We read that there is a statistically significant effect of vaccination (line 24) but it's not clear whether this is BCG vs. PBS, or also subunit vs. BCG, or BCG vs. subunit.  Where was this shown? We read in the abstract that there is a correlation but the figures presented showed that one assay paralleled the results of the other assay (starting line 273).  Wouldn't it be possible to calculate the normalized fluorescence over time (compared to day 0) and also calculate the nomalized CFU over time (compared to day 0), then make a scatterplot to show how well these correlated, in the presence or absence of vaccination?

**In this work, the influence of the vaccination on the decline in fluorescence was analysed using non-linear mixed effects modelling. This statistical analysis methodology is more powerful in identifying relationships in longitudinal data compared to more traditional methodologies. One of the advantages is also that the methodology allows for analysis of non-linear relationships. In this analysis we demonstrated that BCG vaccination led to a higher reduction in fluorescence readout for both fluorophores. P-values are provided in the results section (line 410 and forward). Also, a p-value has now been provided in the abstract for the statistical test of the vaccination effect on fluorescence decline. We have revisited the use of the term “correlates” throughout the text. In addition, the abstract now clarifies that the BCG vaccine has an effect on fluorescence decline and that the CHAD vaccine did not have an effect. (lines 24-27):**

**“The model-based analysis identified that BCG-vaccination reduced the fluorescence readout of both fluorophores compared to unvaccinated mice (p< 0.001). However, vaccination with the novel subunit candidate did not alter the fluorescence decline compared to unvaccinated mice (p> 0.05).”**

4)    Number of experiments, numbers of mice and Figures.  Between the methods and results, it was not clear exactly how many mice were infected / challenged in each experiment and how many datapoints one could expect.  This might be better done with a Table.  For example, on lines 181-183, we read that there were 100 mice, 45 BCG vaccinated, 45 un-vaccinated, 10 ChAd vaccinated.  Figure 1 legends says n = 5, representative of two experiments, but there is no statistical analysis stated here.  Figure 2 legend says data presented are mean of 5 mice per group, average of 2 ears. Can the authors graphically represent the 5 datapoints for each group in 2a, if possible?  I would want to know if the data were Gaussian before using a mean. Assuming a mean is appropriate for 2a and 2b, I would want to know what the statistical analysis was here comparing the curves and whether it was significant, as stated in the abstract.  For 2c and 2d, I think what is shown are the pooled ears from

exp 1 and the pooled ears from exp 2.  The legend says that the graph shows the average of exp 1 and exp 2, I think this should be the averages of each experiment, for clarity.  With pooled data such as these, statistical comparisons do not seem likely to show significance.

**We have added supplementary table S1 to show the experiments and treatments we have performed (mentioned lines 123 and 129) and added additional panels to Figure 1 to show the data from both experiments mentioned. The fixed effects together with random effects in the nonlinear mixed effects modelling used here can handle non-normal distribution and was explored in the analysis.**

5) The authors had two vaccines (BCG and ChAd) and a standard model to evaluate efficacy.  They state in the introduction that there a number of vaccines completing pre-clinical assessment and there is a need to prioritize them.  They compare BCG to no BCG, and ChAd to control, then note in the discussion that ChAd did not work on its own, as that it is expected to boost pre-existing BCG immunization.  **This seemed like a missed opportunity to either compare BCG to ChAd, head-to-head,** or to compare BCG to BCG + ChAd, to look for better protection.  Can this model not be tested for the combination of vaccines that has being studied in human trials, to explore correlations between what is seen with this model and human clinical trial data?

We do have the BCG to ChAd data and the CFU data is shown in Figure 6, which shows no sig diff. The model for RFU also showed ChAd had no impact; we can make that more specific.

**We do have all RFU and CFU data related to Figure 6 to compare BCG v ChAd but it was not plotted since that was not the aim of the experiment, which was rather to investigate the effect of the ChAd vaccine on fluorescence. However, we have now included the additional plots (supplementary figures S10 and S11) to show the RFU and CFU data for the comparisons between BCG and ChAd vaccination (line 397). The model for RFU showed ChAd vaccination had no impact**

6)    The authors mention data not shown at least 3 times in the manuscript. Please verify with the journal on its policy about such statements.

**These were early single experiments superseded by the data in the paper and have been removed.**

**Reviewer #2:**

This is an interesting study using a novel, noninvasive fluorescence monitoring technique to evaluate in mice the feasibility of an intradermal BCG challenge model for assessing in vivo TB immunity. They further assessed with this model in vivo TB immunity induced by 2 different vaccines: BCG and ChAdOx1.PPE15. They do demonstrate that previously BCG-vaccinated mice that are partially protected against an aerosolized Mtb challenge also display more rapid clearance of the fluorescent challenge strain compared with control unvaccinated mice. And they develop a pharmacometric model for analysis of the decline in fluorescence and find evidence that statistically significant covariates for the skin challenge strain clearance included vaccination, baseline fluorescence, added Mtb intranasal challenge and reporter. However, there are some potential problems with the model as described and potential overestimation of the model as a biomarker of protective vaccine-induced immunity.

1) BCG vaccination is given 1 month before the skin challenge. Mice vaccinated with BCG have prolonged persistence of replicating vaccine organisms, lasting up to at least 6-12 months. This may explain why only a small increase in the BCG challenge strain was detected between the challenge day and 2 days post-challenge, and then only reductions in BCG challenge strain load were seen between days 7-21. Maximal innate and/or ongoing effector T cell responses induced by ongoing BCG replication may be active at the time of challenge preventing any meaningful increase in the challenge strain load. These responses may be very different from what is needed to measure long-term vaccine-induced protective memory recall responses against a remote challenge with a pathogen that needs to substantially replicate before disease occurs.

**There is some variation among the different experiments and between control and vaccination groups and we feel it is unlikely that the “small increase in the BCG challenge strain” noted by the reviewer is due to the effects induced by ongoing replication of the vaccine organism, since control groups (without vaccination) sometimes also show little or no increase (Fig 1c) or a decrease (Fig 1e, 2b). The timeline for the biological relevance of the mouse model has been well established over many years, the fine details of the timelines noted by the reviewer are not disputed, but the relevant biological effects measured using the mouse model are an issue. The relevance of the mouse model of TB to human clinical studies remains open to interpretation and was one of the drivers for this work. What we report here is an attempt to move away from the mouse model and take a more experimental medicine approach where difference in the type of candidate vaccines, whether a subunit, or live-attenuated will require different approaches.**

2) In the discussion, on page 25, the authors state that "In our study we have confirmed and extended on the results demonstrating that protection against a skin-based challenge is a predictor of protective vaccine responses in the lung". Although they have seen an association between more rapid skin clearance of the reporter strains and lung protection against Mtb challenge, the current work is far from demonstrating that the skin model alone can be a reliable predictor of mucosal protection. I would remove statements that claim the model is a reliable "predictor" or "correlate" of mucosal protection. At this point I would say the skin model clearance is associated with lung clearance, but whether it can be a direct predictor or correlate are not yet known.

**We have revisited the use of the terms “correlate” and “predictor” throughout the text.**

3) The pharmacometrics model and analyses of covariance are complicated and difficult for this reviewer to adequately evaluate. It would be helpful to have more discussion of what the biostatistical models actually demonstrate and the limitations of the interpretations.

**Please see response to comment 3 from Reviewer 1.**

3) A better schematic of some of the experiments would be helpful. For example, it was difficult for this reviewer to understand without extensive review that some mice were given both a BCG skin challenge and an Mtb IN challenge at the same time which could alter the performance of the skin challenge given alone (in fact, the statistically significant covariance between the skin model responses and added Mtb IN challenge suggest as much).

**We have added Supplementary Table S1 to summarise all the experiments performed in terms of vaccination and challenge with Fluor-BCG and/or H37Rv. The reviewer is correct to note that Fluor-BCG has a vaccine effect against Mtb IN challenge in unvaccinated animals, but this is not seen in BCG-vaccinated animals. These stats have been added to Figure 5.**

5) There are no significant differences shown for direct comparisons of skin load clearance in vaccinated vs unvaccinated responses. For example, no area-under-the curve analyses are reported which would provide a more sensitive endpoint for testing of significant differences between vaccinated and unvaccinated groups. If the model cannot see significant group response differences, it will not be useful for controlled human infection models designed to help select the most promising TB vaccines for advancing beyond phase 1 trials.

**Significant differences have been found. The non-linear mixed effects model shows that BCG vaccination is a significant covariate which leads to reduced fluorescence using both readouts. This is provided in the abstract, in the results and in the discussions. The non-linear mixed effects model we have used describes all the available data from all the experiments simultaneously and indicates a BCG effect compared to unvaccinated mice regardless of the fluorophore used. An area-under-the-curve (AUC) analysis would not provide anything additional to what has been shown. AUC analysis is in general considered less sensitive to detect differences compared to the parametric model-based approach we have used here.**

**Reviewer #3:**

Comments to authors:

The manuscript entitled "A BCG skin challenge model for assessing TB vaccines" by Krishnan et al. is an innovative study that addresses a serious need for the TB community. Current methods to compare new TB vaccines require the comparisons of  BCG-vaccinations to new TB vaccinations followed by natural M. tuberculosis infections, thus requiring large numbers of individuals to enrol in the study. By performing skin challenges with a fluorescent BCG, much smaller numbers of individuals would be needed to compare various vaccine candidates. Thus this work addresses a great need and provides a new tool and approach to assess TB vaccine efficacies. Interestingly, the authors characterize significant differences between the BCG-vaccinated and unvaccinated cellular immune responses that likely correlate with protection. The authors also show a correlation in skin protection with infection in the lung. A number of pointed need to be addressed before publication.

Specific Comments:

1. Since this is the first report of this portable imager, a photograph of the unit as well as an example of the image that is analyzed, would be helpful to the reader. Such a Figure would be more informative than Figure 1A..

**We have added images of the camera and fluorescent ear spots to figure 1.**

2. The "Fluorophore Selection" paragraph result section is unclear. Although reference 19 demonstrates that the ASV tag makes the Turbo635 unstable, it is unclear why this unstable fluorophore is being used. Were the authors trying to distinguish between bacterial growth inhibition from bacterial cell killing? The authors mention msfYFP but fail to define the monomeric super folded YFP and then do not explain the relevance of the operon they have constructed. Can they distinguish actively growing cells from cells in stationary growth? The choice of the promoters is not explained in this section nor in the materials and methods. Did the authors do experiments with each fluorescent gee separately? That would have been useful to explain why they used both. The observation that the highest dose gave reproducible results when compared to lower doses is not surprising. .

**This section (line 247) has been re-written to address the reviewer comments. There are two issues to consider when imaging fluorescent reporters in vertebrates. Firstly, the ability of light to penetrate tissue is a function of its wavelength, with red light above 600nm showing less light absorption by haemoglobin. Secondly, fluorescent proteins are stable, so signals take time to decay after the cell stops producing the fluorophore. To address these issues, we decided to construct a dual reporter strain including an unstable version of a red fluorophore that would ensure tissue penetration and be degraded faster as the bacteria stop growing and die, plus a stable fluorophore protein as an additional marker of bacteria should degradation of the unstable fluorophore take it below detectable levels.**

3.    The "Utilising fluorescence output as a measure of vaccine efficacy in a murine skin challenge model" section provides evidence suggesting that the vaccination controls the infection based on the 5 to 10 fold difference in RLU measurements. This is demonstrated in Fig. 1b and repeated in Figure 2a.a. Figure 2c problematic as the authors report a 10 ro possibly 50-fold differences in CFU's between the unvaccinated and BCG-vaccinated controls at day 2. If I am understanding correctly, the author injected 5 X 106 CFUs in each of 10 ears for 5 mice in each group. Since they pooled the samples they should have had  % X 107 CFUs in both sets of mice assuming 100% recovery. They may be getting that number for the unvaccinated but it is unclear how many significant digits they have as they do not show the data. Interestingly, there is 10 -fold or more less cells in the vaccinated. This might be suggesting that there is a log of killing demonstrable in the first few days. The data would have been more compelling if they had analyzed the ears from individual mice un the experiment.

**We are unclear what the reviewer means with reference to Figure 2c, since on day 2 the CFUs are similar and around the expected 10^7^ for unvaccinated and vaccinated mice. The figure legend does state that these are from pooled samples. We did not assess individual ears in this experiment but that could be done in future work.**

3. **Table 1** would be far more useful for the readers if it included what the molecule and immune cells were recognized by the antibody.

**We have separated out the marker antigens and fluorophore data in a revised Table 1 to make it clearer. The legend to figure S5 details the gating strategy used to define the cell subsets: dermal DCs, dermal langerin+ DCs, monocytes and macrophages.**

4. Did the authors ever analyze the fluorescence in response to TB chemotherapy. This approach could help distinguish killing from growth inhibition. The use of different promoters that express the fluorescence genes could be valuable in future studies. The discussion has several redundancies and should be shortened but this point is worth mentioning.

**This is an excellent idea, but we have not done this yet.**

6. Figure 2A uses measurements that vary from 100,000,000 to 1.000.000.000.  If these are the only ranges measured, would the data be more convincing if reported linearly?  Also, can you recorder 2 or 3 significant digits?  As mentioned, above 2C, would be more useful if reported with 2 or 3 significant digits and if the data had been obtained for individual ears and not pools.  The 2C experiment would need to be reported from three independent experiments since it seems to be reporting an important biological result.

**We chose to plot RFU data on a log scale to be consistent across all the data plots and chose to work with pooled ears rather than individually. Figure 2 reports data from 2 biological replicates, each with 5 mice per group. RFU was measured from each ear treating them as technical replicates and the means taken for analysis. Ears and proximal auricular draining lymph nodes from n=5 mice were pooled and CFUs were quantified at various time points. We did not have the resources to repeat this a third time.**
