# Supplementary material for: A noninvasive BCG skin challenge model for assessing tuberculosis vaccine efficacy
Source: PLoS Biol. 2024 Aug 19;22(8):e3002766. doi: 10.1371/journal.pbio.3002766 (PMC11361749; doi:10.1371/journal.pbio.3002766)
Supplement: Attachment — Submitted filename: Response to reviewers rev2 22Jul24.docx [file pbio.3002766.s017.docx]

**Our responses to the comments and requests are in blue.**

Thank you for your patience while we considered your revised manuscript "A BCG Skin Challenge Model for Assessing TB Vaccine" for publication as a Methods and Resources at PLOS Biology. This revised version of your manuscript has been evaluated by the PLOS Biology editors, the Academic Editor and two of the original reviewers and an additional reviewer.

Based on the reviews, we are likely to accept this manuscript for publication, provided you satisfactorily address the remaining points raised by the reviewers. Please also make sure to address the following data and other policy-related requests.

**a**) We routinely suggest changes to titles to ensure maximum accessibility for a broad, non-specialist readership, and to ensure they reflect the contents of the paper. In this case, we would suggest a minor edit to the title, as follows. Please ensure you change both the manuscript file and the online submission system, as they need to match for final acceptance:

**"A non-invasive BCG skin challenge model for assessing tuberculosis vaccine efficacy"**

**Changed, although the skin challenge part is still invasive.**

**b**) Please note that tuberculosis (the disease) should not be capitalized. This needs to be adjusted in the abstract and throughout the manuscript.

**Done**

**c**) Thank you for providing the names of the foundations that supported the project. However, we also require the grant number (or grant titles if not available)

**Following added:**

**Aeras grant title “Human Challenge Model for TB”**

**BMGF Grant Number OPP1180610**

**d**) You may be aware of the PLOS Data Policy, which requires that all data be made available without restriction: <http://journals.plos.org/plosbiology/s/data-availability>. For more information, please also see this editorial: <http://dx.doi.org/10.1371/journal.pbio.1001797>

Please **supply the numerical values** either in the supplementary files or as a permanent DOI’d deposition for the following figures:

1BCDE

2ABCD

4ABCDEF

5ABCD

6ABCD

S2AB

S3ABCD

S4AB

S6AB

S7AB

S10ABCDEFGH

S11ABCD

**Numerical data for the above supplied as supplementary excel file.**

7 & 8	**Figure legend amended to describe recreation of the plots**

S8 & S9	**Figure legend amended to describe recreation of the plots and supplementary data added.**

NOTE: the numerical data provided should include all replicates AND the way in which the plotted mean and errors were derived (it should not present only the mean/average values).

**e**) Please cite the location of the data clearly in all relevant main and supplementary Figure legends, e.g. “The data underlying this Figure can be found in S1 Data” or “The data underlying this Figure can be found in <https://doi.org/10.5281/zenodo.XXXXX>”

**Done.**

**f**) For figures containing FACS data (Figure 3AB and S5), please provide the FCS files and a picture showing the successive plots and gates that were applied to the FCS files to generate the figure. We ask that you please deposit this data in the FlowRepository (<https://flowrepository.org/>) and provide the accession number/URL of the deposition in the Data Availability Statement in the online submission form

**The FlowRepository gave an error code and no one responded at the helpdesk.**

**FCS files uploaded to Zenodo** [**https://doi.org/10.5281/zenodo.12794251**](https://doi.org/10.5281/zenodo.12794251)**.**

**The gating strategy is shown in Figure S5.**

**g**) Please ensure that your Data Statement in the submission **system** accurately describes where your data can be found and is in final format, as it will be published as written there.

**Revised.**

h) Please ensure that the code generated for the python pipeline is sufficiently well documented and reusable, and that your **Data Statement in the Editorial Manager** submission system accurately describes where your code can be found.

**Deposited at Zenodo as https://doi.org/10.5281/zenodo.12781429**

Please note that we cannot accept sole deposition of code in GitHub, as this could be changed after publication. However, you can archive the code in Zenodo. Once you do this, it will generate a DOI number, which you will need to provide in the **Data Accessibility Statement**.

As you address these items, please take this last chance to review your reference list to ensure that it is complete and correct. If you have cited papers that have been retracted, please include the rationale for doing so in the manuscript text, or remove these references and replace them with relevant current references. Any changes to the reference list should be mentioned in the cover letter that accompanies your revised manuscript.

**Done – no changes made or retracted ms cites as far as I am aware.**

We expect to receive your revised manuscript within two weeks.

To submit your revision, please go to <https://www.editorialmanager.com/pbiology/> and log in as an Author. Click the link labelled 'Submissions Needing Revision' to find your submission record. Your revised submission must include the following:

- a cover letter that should detail your responses to any editorial requests, if applicable, and whether changes have been made to the reference list

- a Response to Reviewers file that provides a detailed response to the reviewers' comments (if applicable, if not applicable please do not delete your existing 'Response to Reviewers' file.)

- a track-changes file indicating any changes that you have made to the manuscript.

NOTE: If Supporting Information files are included with your article, note that these are not copyedited and will be published as they are submitted. Please ensure that these files are legible and of high quality (at least 300 dpi) in an easily accessible file format. For this reason, please be aware that any references listed in an SI file will not be indexed. For more information, see our Supporting Information guidelines:

<https://journals.plos.org/plosbiology/s/supporting-information>

*Published Peer Review History*

Please note that you may have the opportunity to make the peer review history publicly available. The record will include editor decision letters (with reviews) and your responses to reviewer comments. If eligible, we will contact you to opt in or out. Please see here for more details:

<https://blogs.plos.org/plos/2019/05/plos-journals-now-open-for-published-peer-review/>

*Press*

Should you, your institution's press office or the journal office choose to press release your paper, please ensure you have opted out of Early Article Posting on the submission form. We ask that you notify us as soon as possible if you or your institution is planning to press release the article.

*Protocols deposition*

To enhance the reproducibility of your results, we recommend that if applicable you deposit your laboratory protocols in protocols.io, where a protocol can be assigned its own identifier (DOI) such that it can be cited independently in the future. Additionally, PLOS ONE offers an option for publishing peer-reviewed Lab Protocol articles, which describe protocols hosted on protocols.io. Read more information on sharing protocols at <https://plos.org/protocols?utm_medium=editorial-email&utm_source=authorletters&utm_campaign=protocols>

Please do not hesitate to contact me should you have any questions.

Sincerely,

Melissa

Melissa Vazquez Hernandez, Ph.D.

Associate Editor

[mvazquezhernandez@plos.org](mailto:mvazquezhernandez@plos.org)

PLOS Biology

------------------------------------------------------------------------

REVIEWERS' COMMENTS:

**Reviewer #1:**

This paper is much improved. Thank you for your attentiveness to the suggestions of the reviewers.

Some minor points:

1) Page 33, Figure 1 legend mentions panels a to d. But there are now 6 panels. Please update legend.

**Done**

2) Figures 2, 5, 6 show comparable data, especially the CFU plots at the bottom of each. It is a bit difficult to compare across plots with different Y-axes. Would it be possible to present all these plots with the same minimum and maximum, to give the reader a better appreciation of where they are similar and where they differ?

**Figures 5 and 6 have been adjusted to the same Y-axis, but we cannot align the Figure 2 Y-axis with them without clipping data and error bars. We have checked and adjusted line colours and symbols for consistency throughout.**

------------------------------------------------------------------------

Reviewer #3:

The authors have addressed all my concerns adequately. I also think they have done so for the other reviewers. The use of the FluoroBCG and the camera unit make provide important new advances for TB vaccine efficacy studies and I thinks this work merits publication in PLoS Biology

------------------------------------------------------------------------

Reviewer #4:

The authors describe a novel approach to assessing TB vaccine efficacy by describing (i) murine intradermal inoculation with (ii) a recombinant BCG strain with a dual fluorescent reporter and (iii) monitoring fluorescence with a portable imager after vaccination. Overall I found this manuscript to describe a valuable contribution as a Method/Resource.

In this revised version (I did not review the original submission), the authors have attempted to address each of the three reviewers' comments.

However, I find that there is still a lack of clarity in places.

Comments

1. In the Abstract, Introduction and indeed Conclusion I found the focus on human infection models surprising given that what is presented in the paper is in fact an elegant reporter system tested in a mouse model. Reviewer 1 also mentioned this, yet the only change was to add a new line in the Abstract: "Here we were report here (sic) on the characterisation of this system in the mouse model". Indeed, the authors criticise mouse models in the Introduction, "TB vaccine discovery has been hampered by animal models that poorly reflect what happens in humans". I would urge the authors to at least mention positive benefits of development and testing in mouse models, given that is what they present. Indeed, a substantial amount of the work presented is characterisation of murine immune cell populations in the ear. Does this "reflect what happens in humans"?

**We have altered the abstract so that the sentence about mouse models now comes at the start line 12..**

***We report here on the characterisation in mice of a non-invasive BCG skin challenge model for assessing tuberculosis vaccine efficacy. Controlled Human infection models (CHIM) are valuable tools for assessing the relevant….***

**A sentence has been added line 51 about what has been learnt from the mouse model of tuberculosis:**

***We have learnt much about the pathogenesis and immunology of tuberculosis using the mouse model.***

**How the immune response in the skin reflects that in the lungs is discussed line 453. Indeed BCG and many other vaccines are given intradermally to generate protective immune responses in other body compartments.**

2. I found the recombinant Fluor-BCG, with the dual reporters and panCD system for plasmid stability, to be very elegant. I would add specific mention of 'Fluor-BCG' to the abstract.

**Done**

3. Lines 497-516: Could the authors improve the language in this section, and avoid the multiple uses of vague, non-quantitative phrases such as: "a slightly more initial rapid decline", "a more rapid initial decline", "a slightly more rapid", "somewhat milder initial decline", "a slightly steeper decline", etc.

**Removed**

4. Line 262: "Quantification showed a 10-fold decrease Turbo635ASV RFUs compared to YFP (Figure S2)". Should this not say "10-fold less Turbo635ASV RFUs" compared to YFP? A 'decrease' suggests it started off high and then reduced compared to YFP; that is not what is shown in Fig S2.

**Corrected.**

5. Line 273-74: "The high dose inoculum provided the optimum signal to noise ratio and was well tolerated in mice with no adverse effects (Supplementary Fig S2)". Based on the data presented in the manuscript, it appears only the YFP "provided the optimum signal to noise ratio", and I suggest this is clarified.

**Changed to specific for YFP.**

6. Line 251: Add mention of Fluor-BCG "we decided to construct a dual reporter strain, Fluor-BCG,…"

**Added**

7. Line 307: "To confirm movement of BCG from the ear…". This should be "To confirm movement of Fluor-BCG from the ear…"

**Added**

8. Line 356: "Fluorescence readout from the skin acts as a relates to protective immunity in the lung". This needs to be reworded.

**Done: *Fluorescence readout from the skin relates to protective immunity in the lung***

9. Figure S4: There is an error in the legend, where "Vaccinated_FluorBCG" is represented by circles in the legend, but graphed as squares in S4(b).

**We have checked and adjusted line colours and symbols for consistency throughout.**
